# Patella sleeve fracture injury: a case report

**DOI:** 10.4314/gmj.v55i1.15

**Published:** 2021-03

**Authors:** Reuben K S Ngissah, Nana K Gyeke-Boafo, Lawrence K B Awere-Kyere

**Affiliations:** 1 Department of Surgery, Greater Accra Regional Hospital, P. O. Box 473 Accra, Ghana

**Keywords:** Patella, sleeve, fracture, surgical treatment

## Abstract

Patella sleeve fracture is a rare injury that occurs in children and is characterized by an avulsion of extensive sleeve of cartilage and periosteum with small bony fragments, usually from the inferior pole of the patella. It is important to make this diagnosis promptly and act accordingly, because a delay or misdiagnosis will result in severe permanent disability to the affected child. A case of this injury is presented to create awareness among physicians, especially front-liners within the medical community in Sub-Saharan Africa, where such an injury has been rarely reported in the literature. The presentation, evaluation, management and outcome over a six months period is being presented.

## Introduction

Patella sleeve fracture is a rare injury that occurs in children and should not be missed. This injury may be easily overlooked on plain radiograph resulting in misdiagnosis or delay in its management. A high Index of suspicion is required to make the diagnosis, taking into consideration the mechanism of injury, clinical examination findings and the subtle observations on plain radiograph. Once the diagnosis has been established, treatment must be prompt to restore anatomy and to return the patient to the preinjury state without any disability.

## Case Report

**Figure 1:**
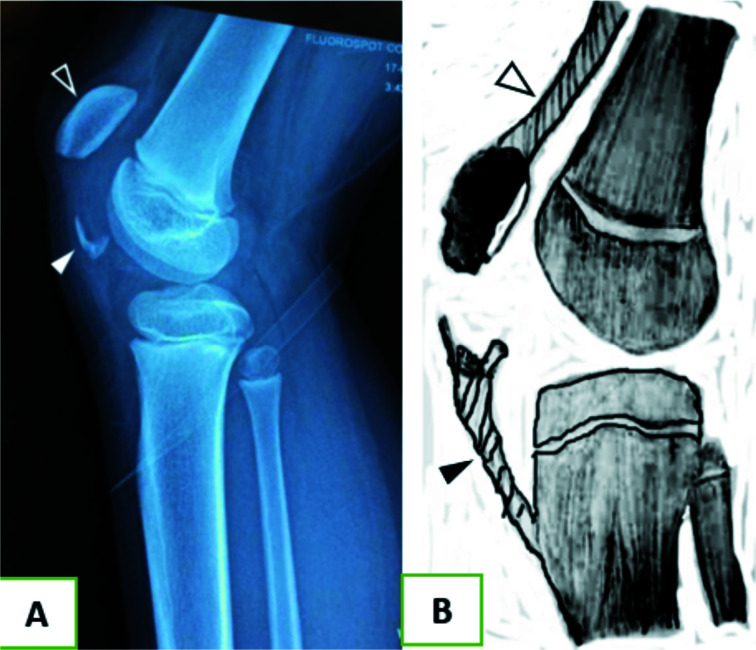
A: Plain x-ray of the patient with inferior periosteal sleeve of the patella, appearing as fleck of bony tissue (closed arrowhead) and high riding patella, patella alta, open arrowhead). Figure 1-B: Schematic representation of sleeve fracture, with quadriceps tendon (open arrowhead) and patella tendon (closed arrowhead)

An eight-year-old girl presented to Greater Accra Regional Hospital with a day history of sudden onset of severe pain in the right knee after she jumped a flight of stairs while descending. This was associated with inability to bear weight. She had no other injury. Physical examination findings revealed a little girl who was in severe pain; grossly swollen and tender right knee with marked restriction in motion, and inability to perform a straight leg raise. All other examination findings were normal. A plain x-ray of the right knee in the lateral view, showed patella alta, with flecks of bone around the inferior aspect of the patella (Figure 1). A diagnosis of patella sleeve fracture was made.

**Figure 2:**
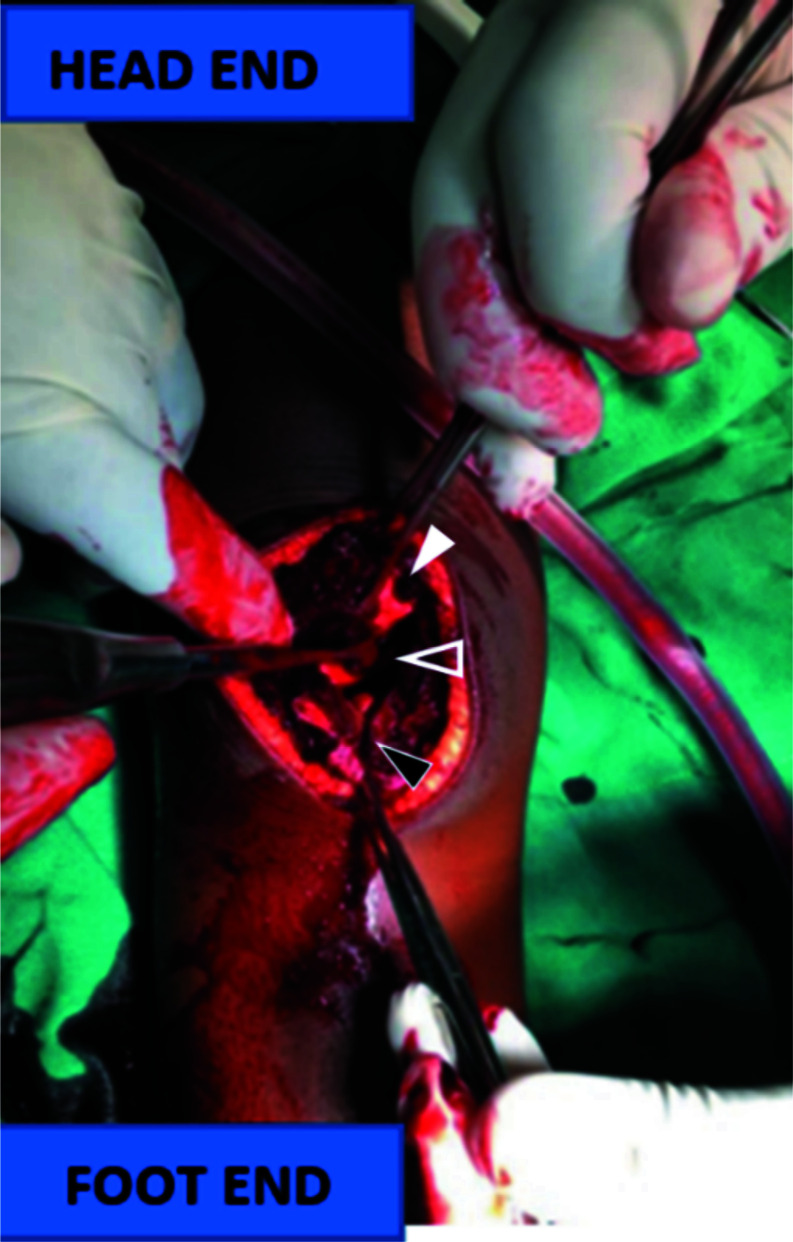
Intraoperative view showing bony patella (closed white arrowhead), gap between bony patella (open arrowhead) and inferior patella sleeve (closed black arrowhead).

She was subsequently prepared for surgery. Intraoperative findings, were an avulsion of the inferior periosteum and cartilage of the patella, with a small bony fragment as well as torn medial and lateral retinacula (Figure 2).

**Figure 3:**
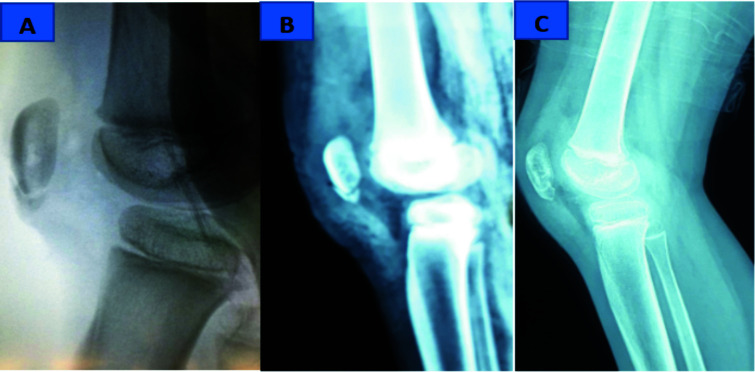
A- Intraoperative fluoroscopic view; B- Immediate post-operative view; C- 3 Months post op, showing complete healing of sleeve fracture

She had repair of the avulsion injury with an absorbable intra-osseous anchor suture and repair of the torn retinacula with Ethibond suture. The reduction was confirmed intraoperative with fluoroscopy (Figure 3A) and was found to be satisfactory. Immediate post-operative x-ray of the right knee also revealed excellent reduction of the avulsed segment (Figure 3B).

At three months, patient was bearing weight and had started performing basic activities of daily living on her own. Plain x-ray of the knee in the lateral view showed complete healing of the avulsion fracture, characterized by complete obliteration of the fracture line (Figure 3C).

**Figure 4:**
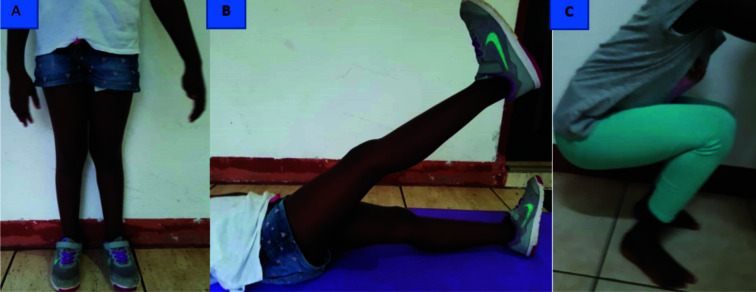
At six months; A: Child standing without support; B: Performing a straight leg raise; C: Squatting

At 6 months, she had resumed all activities of daily living and was capable of squatting and performing a straight leg raise (Figure 4).

### Ethical consideration

This case report received institutional permission from the Greater Accra Regional Hospital, Accra, Republic of Ghana. Informed consent was also given by the parents of the child

## Discussion

Patella fractures are rare in children, because the patella which is mainly cartilaginous and radiolucent begins ossification at about 5 to 6 years of age.^1^, ^2^ Patella sleeve fracture, although a rare variant of patella fracture, appears to be the common type of patella fracture in children and occurs between the ages of eight and 16 years.^1^ It occurs more frequently in boys than girls at a ratio of approximately 3:1, respectively.^3^ It involves the separation of the thick sleeve of cartilage with a small fragment of bone, commonly from the inferior pole of the patella as depicted in Figure 1B. It occurs when the quadriceps contracts suddenly and forcefully against a flexed knee^4^, this usually follows a non-contact mechanism that involves jumping, such as basket-ball or trampolining. Clinically the patient presents with, a painful swollen knee of sudden onset with inability to bear weight. Physical examination will reveal a swollen tender knee joint, with a palpable gap and patella alta (high riding patella), and inability to perform a straight leg raise.

Plain x-ray of the knee, may be deceptive to the unwary and inexperienced clinician, as it will only show a small, often insignificant appearing fragment of bone (Figure 1 A). However, an extensive sleeve of cartilage also, is pulled from the patella (Figure 1B), making this a significant injury despite its benign radiographic features as portrayed by the case presented.

Ultrasound and MRI are very useful in detecting avulsed fragments that are devoid of bony tissue (made up of periosteum and cartilage only) 5 6, hence making it difficult to interpret x-ray findings. In this index case, routine ultrasonography nor MRI of the knee were not done because, the diagnosis was entertained after tying the history with x-ray findings, of patella alta and fleck of bony tissue at the inferior aspect of the patella.

In a relatively resource constrained environment requesting for an MRI may delay the diagnosis because of delay in performing and reporting on the images; however, MRI is a must if the avulsed fragment is devoid of bony tissue as mentioned earlier.

Clinching the diagnosis of patella sleeve fracture is very critical, because failure to do so, will cause the displaced bone-forming tissue to continue to grow leading to ectopic bone formation and subsequent permanent morbidities such as instability of the knee, reduced extension, quadriceps wasting and weakness.^7^

Some differential diagnosis to be entertained include; Sinding-Larsen-Johansson disease-a chronic overuse injury characterized by pain and tenderness at the inferior pole of the patella^3^; Osgood Schlatter Disease, also a chronic overuse injury rather than a traumatic event characterized by pain and tenderness at the point of insertion of the patella tendon at the tibia tubercle and Tibia Tubercle avulsion fracture- an acute injury characterized by knee pain and swelling, occurring in older adolescents during the transitional phase of physeal closure just before completion of growth.^9^

Treatment of patella sleeve fracture is mainly surgical, and has to be prompt to forestall all the complications that have been mentioned above. It involves open reduction and holding the reduction with anchor sutures, interfragmentary screws or Kirschner and cerclage wires^10^, ^11^. The use of Kirschner and cerclage wires in holding the reduction may necessitate a second surgery for its removal after healing has occurred. The presented case, was saved from a second surgery because she had the repair been done, with a bioabsorbable intraosseous anchor suture and heavy Ethibond sutures. In rare situations, and when the displacement is less than 2mm, conservative management with a hinged knee brace that is initially kept in extension and gradually flexed over 6 weeks will suffice.^12^ Generally, the prognosis is good to excellent with respect to function and activity, when identified early and prompt surgical treatment performed^13^ as demonstrated in the presented case.

## Conclusion

Patella sleeve fracture is a diagnosis that requires a high index of suspicion, especially in the skeletally immature patient who is presenting with acute knee pain of sudden onset, preceded by a non-contact sport or activity which involves sudden jumping. It is crucial to arrive at this diagnosis because failure to do so will lead to severe and permanent disabilities.
